# Case report: Evisceration of abdomen after blunt trauma

**DOI:** 10.1016/j.ijscr.2020.05.037

**Published:** 2020-06-01

**Authors:** Arwa H. Ibrahim, Adel J. Osman, Mosab A. Alarfaj, Areej M. Alzamil, Munirah A. Abahussain, Hanan Alghamdi

**Affiliations:** a College of Medicine, King Fahd Hospital of the University, Imam Abdulrahman Bin Faisal University, Saudi Arabia; b King Fahd Hospital of the University, Imam Abdulrahman Bin Faisal University, Department of Surgery, Saudi Arabia

**Keywords:** Abdominal, Evisceration, Blunt, Trauma, Pancreatic, Transection

## Abstract

**Introduction:**

Abdominal evisceration is uncommon after blunt abdominal trauma; therefore, it warrants urgent laparotomy.

We report a young adult male who sustained multiple injuries due to a high impact mechanism resulting in blunt abdominal injury and underwent numerous laparotomies.

**Case report:**

In a high-speed motorcycle accident, a twenty-six-year-old male sustained a direct, blunt injury to his abdomen, which resulted in a right hemothorax, perforation of the stomach, and small bowel. Multiple mesenteric vessels tear, a retroperitoneal hematoma, liver, and pancreatic injury. The abdominal wall split transversely, extruding intact bowel. After resuscitation, according to the ATLS protocol, the patient underwent eight laparotomies for damage control. After 45 days in the Surgical Intensive Care Unit, then 11 days in the surgical ward, he was discharged in a satisfactory condition. Eight months later, he was admitted electively for ileostomy reversal, which was uneventful.

**Conclusion:**

Patients with high trauma mechanisms have high mortality and morbidity rate. Blunt injury with eviscerated abdominal contents requires prompt, expeditious, and timely intervention, particularly at the initial operative intervention with damage control procedures, both prompt management and structured approach, were tailored depending in the magnitude of the injury. A multidisciplinary approach is mandatory throughout the period of treatment until recovery and rehabilitation.

## Highlights

• Patients with severe mechanisms of injury have a high mortality and morbidity.
• Severe blunt injury to the abdomen requires timely intervention, especially if there is evisceration.
• A multidisciplinary team approach is mandatory.

## 1 Introduction

Traumatic injury is the leading cause of emergency department (ED) visits, hospital admission, temporary or permanent disability, and death [1]. Motor vehicle accident (MVA) is one of the significant causes of injury-related deaths accounting for more than 80% of all trauma admissions in Saudi Arabia [2]. Moreover, in trauma victims, the abdomen is the third most common injured region [3]. Susceptibility of the abdomen to injuries could be attributed to the minimal protection for underlying organ by bones [4]. Abdominal organ evisceration is uncommon to be encountered, particularly after blunt abdominal trauma, therefore it warrants urgent laparotomy [5]. This work has been reported in line with the SCARE criteria [6].

## 2 Case report

Twenty-six years old Saudi male, a victim of a high-speed motorcycle accident, where he lost control and hit an iron roadblock. He sustained a direct, blunt injury to his abdomen. He was wearing personal protective equipment. The rider was brought to ED by the Red Crescent ambulance with severe eviscerated abdomen transversally and de-gloving of the chest wall (Picture 1).

**Picture 1:**
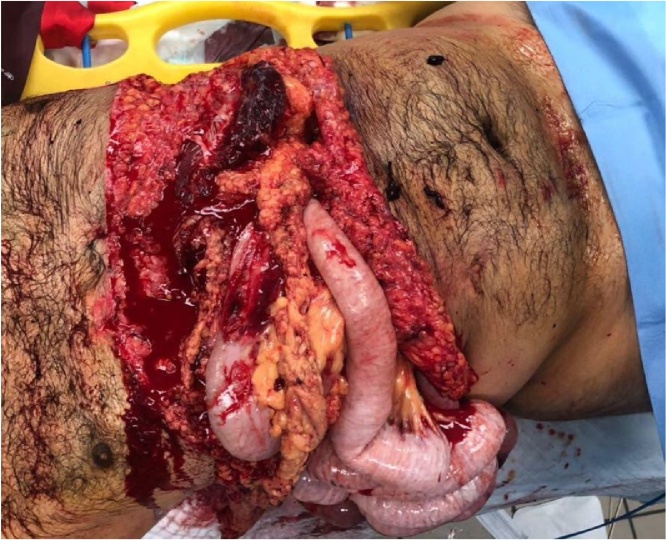
Eviscerated bowel segments after trauma.

He was immediately intubated in critical status and shifted to the operating room for exploration as a damage control surgery after the primary survey.

The patient underwent eight exploratory laparotomies for damage control open abdomen, packing, revision, repair, lavage, and definitive surgery.

First exploration; at presentation (damage control surgery by Trauma and vascular team); Hgb: 3.2 g/dL. Plt: 74 k/ul. Blood pressure was maintained by blood and components transfusion + inotropes.

Eviscerated abdomen transversally where muscles were avulsed from the ribcage to approximately mid-axillary line and exposed the ribs bilaterally.

**Fig. 1:**
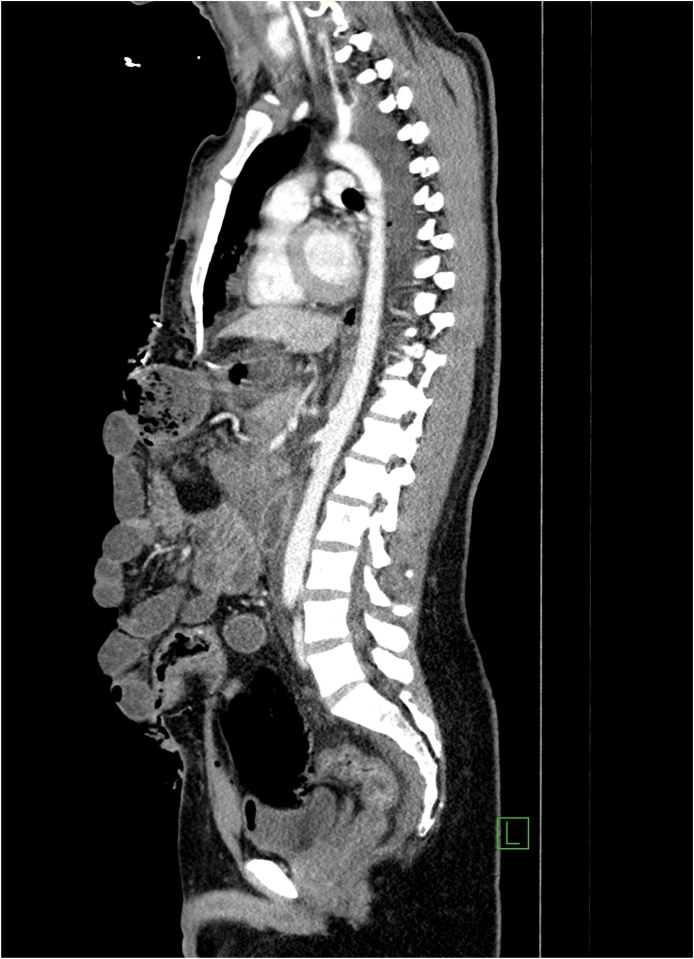
Sagittal enhanced CT of the abdomen showing anterior abdominal wall defect with multiple bowl loops evisceration.

**Fig. 2:**
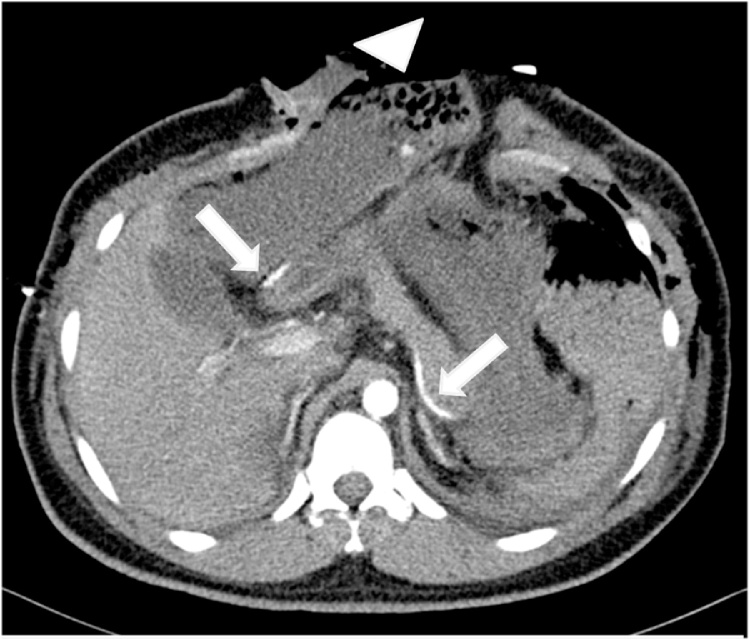
Axial Enhanced CT of the abdomen post explorative laparotomy shows; A: surgical suture lines along the greater curvature of the stomach (bold arrows),Anterior abdominal wall defect with part of the stomach herniating through it (arrowhead).

**Fig. 3:**
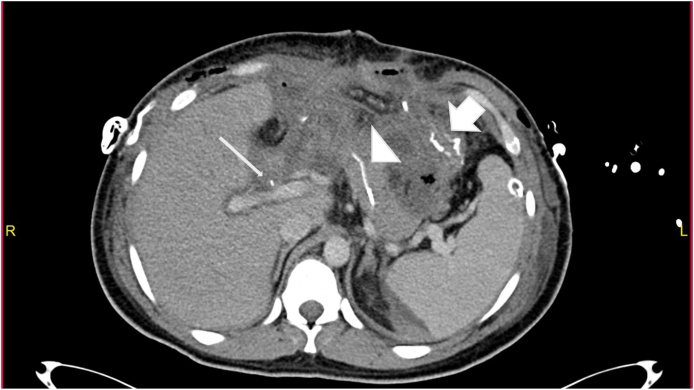
Axial enhanced CT scan of the abdomen post whipple procedure showing; A: gastrojejunostomy (Bold arrow), B: pancreaticojejunostomy(arrowhead), C: Choledochojejunostomy (thin arrow).

Through a midline incision:

• Perforation of the stomach at the greater curvature about 2 cm managed by primary repair with a linear stapler.
• Multiple mesenteric vessels' tears ligated.
• Perforation of small bowel at about 100 cm from the ileocecal junction, resected by stapler without anastomosis.
• Large central expanding retroperitoneal hematoma (zone 1), exploration was carried out by Mattox then Cattel-Braasch maneuver with supra celiac aorta clamping and evacuation of the hematoma.
• Minor bleeding points controlled and packing done.
• Grade one liver injury identified in the right and left lobe.
• The abdomen was left open. Then the patient was shifted to the crash area where secondary survey and PAN CT done, then admitted to SICU.
• PAN CT report:
  - Postsurgical changes noted in the form of intraperitoneal and extraperitoneal packing with multiple intra-and extraperitoneal hematomas.
  - Free fluid and air pockets associated with surgical emphysema.
  - Diffuse small-bowel loops mucosal hyperdensity could be a hematoma.
  - Liver hypo-density in segment IV likely to be laceration associated with periportal edema.
  - Peripancreatic free fluid is noted, which can be attributed as postsurgical change (Fig. 1, Fig. 2, Fig. 3).

Second exploration; Day 2 post-admission (Trauma team); Hgb: 5.6 g/dL. Plt: 48 k/ul.

• Previously resected small bowel segment 100 cm from the ileocecal junction anastomosed side – side.
• Another area of small bowel 70 cm from duodenojejunal junction was found dusky, resected and anastomosed side – side.
• Multiple serosal tears at transverse colon and cecum, primarily repaired.
• Spillage of bile identified in the retroperitoneum.
• Partial transection of the neck of the pancreas (main duct intact).
• Kocherization of duodenum showed approximately 2 cm perforation in 3rd part of the duodenum, and A catheter was inserted for drainage as a controlled fistula.
• Changing of abdominal packing carried out and hemostatic agent applied to control oozing (Picture 2).

**Picture 2:**
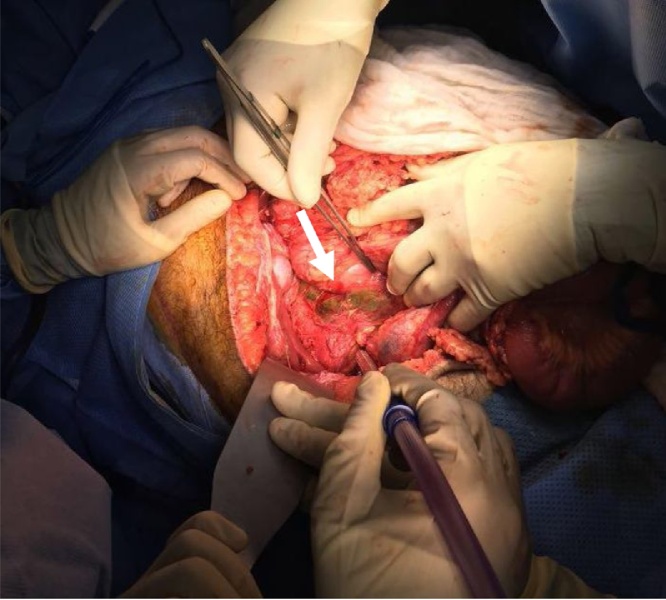
Green discoloration in retroperitoneal area after kocherization injury between 2nd and 3rd part of duodenum.

During both first and second exploration, the patient was kept with an open abdomen (No closure of skin and fascia). We used an Opsite sandwich technique to cover the abdominal cavity, which is made of two Opsite transparent waterproof sheets sandwiched together with large lap gauze in-between.

Third exploration; Day 5 post-admission (hepatobiliary team); Hgb: 8.1 g/dL. Plt: 92 k/ul.

• Exploration and removal of all abdominal packing with a meticulous examination of bowel from the stomach to the rectum.
• A narrowed segment of the transverse colon, which was repaired in the second exploration, was resected and anastomosed side-side with a linear stapler.
• The duodenal injury was repaired primarily in two-layers PDS 3–0 and prolene.
• Two tension sutures of prolene 2–0 applied to both corners of the pancreas.
• Hemostasis and irrigation were done, with four drains inserted.
• Closure of the incised fascia and abdominal skin.

Fourth exploration; Day 10 post-admission (hepatobiliary team), on the fifth day after the third exploration, general patient condition suddenly deteriorated, bowel contents were seen in the abdominal drains from the surgical wound, exploration showed devitalized duodenum in addition to the right-side colon to the mid-transverse colon. Therefore, the patient underwent a Whipple procedure and extended right hemicolectomy with ileostomy creation.

Fifth – eighth explorations were for abdominal lavage.

The patient was admitted to the SICU for a total of 45 days for close monitoring. Afterward, he was transferred to the ward for 11 days and continued to have an uneventful recovery until he was discharged in a satisfactory condition.

Eight months later, the patient was electively admitted to the hospital for ileostomy reversal, where he underwent uneventful exploratory laparotomy with Ilio-sigmoid anastomosis and skin graft of the abdominal wound.

Currently, the patient is on regular outpatient clinic follow up for the past one year in a satisfactory condition with complete resolution of his intra-abdominal injuries.

## 3 Discussion

Blunt abdominal trauma resulting in gastric perforation is not the usual presentation in which the incidence of gastric rupture is very low 0.02–1.7% [7]. Variety of trauma factors influencing the gastric injury include; site and location of the injury, time of the last meal and seat belt use [8]. The surgical management in small or single lesions is comprised of debriding the wound and simple suturing; On the other hand, partial gastrectomy might be indicated in cases with extensive damage to the stomach [7].

According to *Rodríguez-Hermosa*; such gastric lesions are associated with injury to other organs including the liver, the spleen, the pancreas, the duodenum [7].

Similar to gastric perforation, duodenal rupture is extremely rare to be found after blunt abdominal trauma accounting for only 0.2% (208 patients) in a retrospective study analyzing trauma database of 103,864 patients [9] moreover, those types of duodenal injury range from intramural hematoma to a complete duodenal transection and devascularization [9]. Management includes primary closure (duodenorrhaphy) or resection and anastomosis, which is the method used in most cases [10]. Other methods of surgical management include duodenal diverticulation, pyloric exclusion, pancreaticoduodenectomy, or controlled duodenal fistula [10].

Although gut injury in blunt abdominal trauma is uncommon; however, when it occurs, small and large bowel injuries are the most common to be involved [11]. According to *Raharimanantsoa*, in his study, the incidence of blunt bowel or mesenteric injury ranges from 1 to 5% after blunt abdominal trauma [11]. While the mortality rate reaches up to 18% [12]. The serosal tear is considered the most common colonic injury in blunt abdominal trauma [13]. In similar cases, it was recommended to perform a simple resection and anastomosis rather than colostomy in a recent metanalysis [14]. Resemblance can be found between our case management and to what was reported in *Hardcastle* paper regarding Serosal tear management [15]. On the other hand, the cases in *Dongo* research were managed mainly by hemicolectomy or colostomy [13].

The liver is likely to be injured in blunt trauma due to its size, high vascularity, anterior anatomical position, delicate Glisson's capsule, fragile parenchyma, and the less protective measures taken; the more severe the resulting damage [16]. According to *Renson A et al.*, the mortality rates after liver injury are associated with the Grade of injuries; Low-grade (AAST I-III) accounts for 9.9%–10.4% mortality, while in AAST IV, V and VI, the mortality increases sharply, reaching 27.9%, 64.8%, and 94.9%, respectively [16].

The management of blunt traumatized hepatic lesions shifted towards non-surgical role and observation by radiological methods like Computed tomography scan and magnetic resonance imaging, especially those lesions graded I and II like in this case [17,18].

While less than 2% of injuries to the pancreas are caused by a blunt abdominal injury, mainly due to its anatomical location as a retroperitoneal organ [19]. Despite this fact, pancreatic injury accounts for a high percentage of morbidity and mortality, with morbidity rates ranging between 45% and 60%, and mortality rates ranging between 23.4% and 30.2% [20].

Management of pancreatic trauma depends on the Grade of the injury, Grade I and II usually treated with simple drainage [20]. (Table 1) a study was conducted in the period between 1998–2004 mentioned that out of 23 pancreatic injuries, 22 patients were managed by simple drainage [21]. Grade III managed by distal pancreatectomy, grade IV and V are difficult to manage; in some cases, pancreaticoduodenectomy (Whipple procedure) might be performed [19].

**Table 1:** American Association of the Surgery of Trauma classification of pancreatic trauma-Organ Injury Scale (AAST-OIS).

Grade	Type of injury	Description of injury
I	Hematoma	Minor contusion without ductal injury
	Laceration	Superficial laceration without ductal injury
II	Hematoma	Major contusion without ductal injury or tissue loss
	Laceration	Major laceration without ductal injury or tissue loss
III	Laceration	Distal transection or pancreatic parenchymal injury with ductal injury
IV	Laceration	Proximal transection or pancreatic parenchymal injury involving the ampulla
V	Laceration	Massive disruption of the pancreatic head

In this case, the patient had retroperitoneal hematoma at zone I, which was defined in 1982 by *Kudsk* and *Sheldon* as a collection of blood in the central- medial area which is superiorly bound by the central diaphragm, medial borders of the psoas muscles at its sides and inferiorly the pelvis [21]. The study, which conducted in 1992, revealed that patients with zone I hematoma account for 14% of the total of 233 patients, while the mortality rates associated with such hematomas range from 18% to 31% [22,23]. The most common etiologies of hematoma in the zone I are injuries to major abdominal vessels, especially mesenteric vessels, pancreas, and duodenum [11,21,22]. The general protocol in managing an unstable patient with retroperitoneal hematoma is to go for emergency exploration, like what was done in this case, which will help in early detection and control of the source of bleeding, resulting in decreased incidence of morbidity and mortality [21,22,23].

## 4 Conclusion

Patients with severe injury mechanisms have high mortality and morbidity rates. Severe abdominal blunt injury with evisceration requires prompt, expeditious, and timely intervention, particularly during the initial operative intervention with damage control procedures using the open abdomen technique, both prompt management and structured approach, are tailored to each patient depending in the magnitude of the injury. A multidisciplinary team approach is mandatory throughout the period of treatment until recovery and rehabilitation.

## Declaration of Competing Interest

No conflict of interest to declare.

## Sources of funding

No funding received.

## Ethical approval

Our research type is case report, due to current circumstances our IRB approval currently not accessible.

## Consent

Written informed consent was obtained from the patient for publication of this case report and accompanying images. A copy of the written consent is available for review by the Editor-in-Chief of this journal on request.

## Author contribution

**Arwa H. Ibrahim**: literature review, written first draft of the manuscript, reviewing and editing.

**Adel J Osman**: provided research materials, patient recruitment and management, obtaining photos, reviewing and editing the manuscript.

**Mosab A alarfaj**: provided research materials, patient recruitment and management, obtaining photos, reviewing and editing the manuscript.

**Areej M alzamil**: literature review, written first draft of the manuscript, reviewing and editing.

**Munirah A Abahussain**: literature review, written first draft of the manuscript, reviewing and editing.

**Hanan Alghamdi**: reviewing the manuscript.

All authors participated in general discussions, critically reviewed and approved the final draft and are responsible for the content and similarity index of the manuscript.

## Registration of research studies

NA.

## Guarantor

King Fahd Hospital of the University, Imam Abdulrahman Bin Faisal University, Dammam, Saudi Arabia.

## Provenance and peer review

Not commissioned, externally peer-reviewed.
